# The significance of building behavior in the evolution of animal architecture

**DOI:** 10.1111/1440-1703.12309

**Published:** 2022-03-24

**Authors:** Shoko Sugasawa, David J. Pritchard

**Affiliations:** ^1^ Centre for Biological Diversity, Harold Mitchell Building, School of Biology University of St Andrews St Andrews UK; ^2^ Biological and Environmental Sciences University of Stirling Stirling UK

**Keywords:** animal architecture, animal cognition, building, construction behavior, ethology

## Abstract

Animals make a diverse array of architectures including nests, bowers, roosts, traps, and tools. Much of the research into animal architecture has focused on the analysis of physical properties such as the dimensions and material of the architectures, rather than the behavior responsible for creating these architectures. However, the relationship between the architecture itself and the construction behavior that built it is not straightforward, and overlooking behavior risks obtaining an incomplete or even misleading picture of how animal architecture evolves. Here we review data about animal architectures broadly, with a particular focus on building by birds and social insects. We then highlight three ways in which a better understanding of building behavior could benefit the study of animal architecture: by clarifying how behavior leads to physical properties; by examining the costs and benefits of building behavior; and by determining the role of learning and how this interacts with selection on behavior. To integrate questions about building behavior alongside those about architectures, we propose a framework inspired by Niko Tinbergen's four questions, examining the mechanistic, ontogenetic, phylogenetic, and functional basis of animal building. By integrating the study of behavior and architecture across levels of analysis, we can gain a more holistic view of the behavior‐architecture interactions, and a better understanding of how behavior, cognition, and evolution interact to produce the diversity seen in animal architecture.

## Graphical abstract

Animals make a diverse array of architectures including nests, bowers, and tools. The relationship between the architecture itself and the construction behavior that built it is not straightforward and overlooking behavior risks obtaining an incomplete or even misleading picture of how animal architecture evolves. In this review, we highlight the ways in which a better understanding of building behavior could benefit the study of animal architecture.

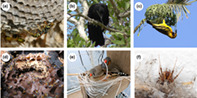

## 1 INTRODUCTION

**FIGURE 1:**
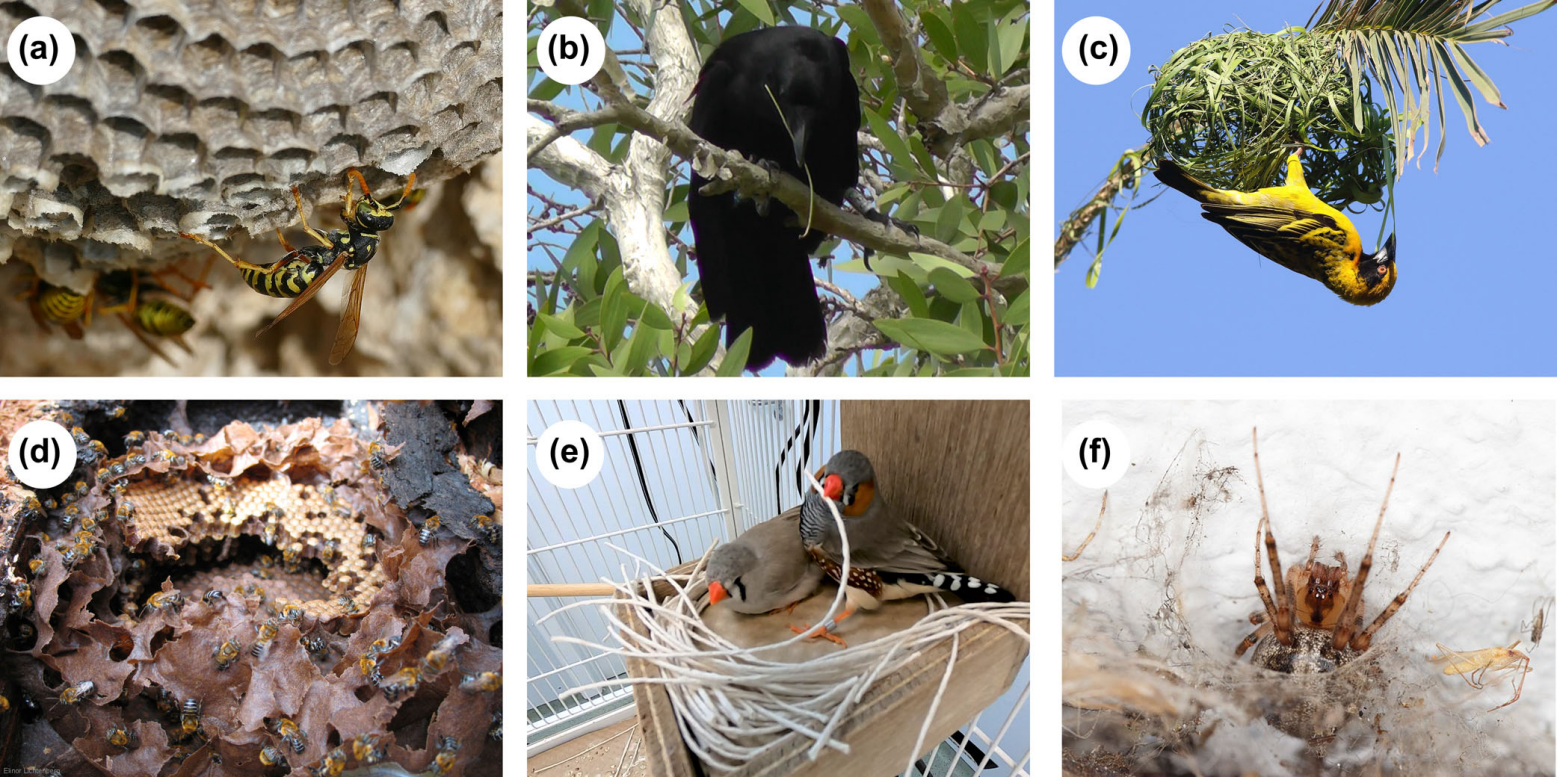
Diverse architectures built by animals. (a) Paper wasps, such as European paper wasps (*Polistes dominula*), build a nest from a mixture of fibers and saliva. (b) New Caledonian crows (*Corvus moneduloides*) make and use tools to capture prey. (c) Village weavers (*Ploceus cucullatus*) make a nest by weaving grass strips. (d) Stingless bees (*Melipona scutellaris*) build brood cells for eggs. (e) Zebra finches (*Taeniopygia guttata*) use various materials to build nests in captivity. (f) Missing‐sector orbweaver spider (*Zygiella x‐notata*) learn to move their webs in response to various factors. Photos by (a) Bernard Dupont; (b) Pedro Barros da Costa; (c) Alandmanson; (d) Elichten; (e) Shoko Sugasawa; and (f) Dariusz Kowalczyk. Photos (a), (c)–(f) are licensed under the Creative Commons copyright license. (b) is reused with permission from Sugasawa et al. (2017) [Color figure can be viewed at wileyonlinelibrary.com]

Many animals build architectures such as nests, traps, bowers, and tools (Hansell, 2005). Some of these architectures are remarkably intricate, some colorfully decorated, and some hundreds of times larger than the builders themselves (Tello‐Ramos et al., 2022; Sugasawa et al., 2021). Although the term architecture is usually reserved for buildings and shelters built by humans, here we use it to mean a wide range of physical structures built by non‐human animals for foraging, breeding, and sheltering (Figure 1). This architectural diversity, covering nests, tools and other animal‐made structures, rivals the diversity seen in the colors and morphologies of animals themselves and, like these organismal traits, animal architectures are seen as products of selection. Unlike these organismal traits, however, animal architectures are not the direct product of genes and development, but are realized via the behavior of their builders. The type of behavior performed by the builders (e.g. digging dirt, piling up twigs, weaving grass) defines the properties of the architectures, including both physical attributes such as size, shape, and materials, as well as more task‐specific features such as strength, flexibility, or thermal characteristics. This key role for behavior in linking selection on the genes of the builders to the properties of animal architectures has led to animal construction being considered as prime examples of extended phenotypes (Dawkins, 1982).

**FIGURE 2:**
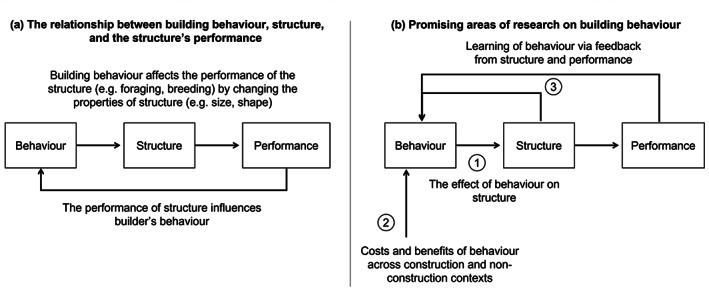
The relationship between the builder's behavior, animal architecture, and the architecture's performance. (a) While animal architectures are the products of building behavior, the performance of the architecture and the resulting fitness consequences depend on their properties. The central role of the structure in determining performance has led to research focusing on the physical properties of architectures while relatively neglecting behavior. (b) The relationship between building behavior and the resulting animal architectures is more complicated that a simple one‐to‐one correspondence: (1) The link between behavior and structure is currently poorly understood and similarities in structure might hide differences in behavior; (2) Selection on building behavior is not just based on the performance of the structure but also on the costs and benefits of the behavior itself; (3) Building behavior is not just the product of genetic evolution but can be shaped by both asocial and social learning

For many key behaviors studied by behavioral ecologists, such as foraging and provisioning, selection on these behaviors is directly linked to the properties of the behavior. Reproductive success or fitness proxies, such as net energy rate, are compared with behavioral measures such as distance traveled or when an animal leaves a patch. For construction behavior, on the other hand, selection only acts via the function of the architectures the animal creates (Figure 2a). Most research on animal architecture has therefore focused on these functional properties of architectures, and how variation in these properties affects fitness. Chimpanzees, *Pan troglodytes*, in Republic of Congo, for instance, add a brushed tip on an end of termite‐fishing tools by fraying the end. When tested by scientists, these brushed‐tip tools were more efficient for catching termites than tools with a plain end (Sanz et al., 2009). Furthermore, chicks of tree swallows, *Tachycineta bicolor*, raised in nests with more feather lining grew up larger (Dawson et al., 2011), while nestlings of lesser kestrels, *Falco naumanni* survived longer in deeper nests, potentially because they could hide better from predators (Sarà et al., 2012). Although these differences in the form of animal architectures are assumed to be the result of differences in building behavior, this line of research takes an approach more similar to functional morphology than behavioral ecology, examining how subtle variation in the form (e.g., material, morphology) of the built architecture relates to differential success in survival or reproductive success (Hansell, 2000, 2005).

While selection might depend on the properties of animal architectures, these properties are still the results of building behavior, and changes in these properties can only be realized by animal changing their behavior (Figure 2a). If the physical properties of animal architecture are under selection, therefore, this must be achieved by selection shaping building behavior. Despite this crucial role for behavior, and despite the long history of researchers examining the form and function of animal architecture, the behavior part of “construction behavior” remains poorly understood. In part this is because studying construction behavior itself is difficult. While animal architectures are inanimate objects that can be easily and precisely measured, behavior is messy, transient, and can be difficult to directly observe or quantify. To understand how animal architectures acquire their properties and how selection shapes these properties, however, it is not sufficient to only study inanimate architectures. It is also necessary to directly study building behavior. In this review, we discuss several promising areas in which studies of building behavior are providing a new perspective on how animal architecture evolves. As both authors have worked primarily on birds as their research system, and there is a large body of existing literature on avian architecture, many of our examples come from research using birds. Our message, however, extends far beyond the phylogenetic boundaries. By building on these areas we describe below, we can broaden the study of animal architecture beyond just questions of form and function, examining how ecology, cognition, and evolution shape the architectures animals build.

## 2 HOW DOES VARIATION IN BUILDING BEHAVIOR MAP ONTO VARIATION IN ARCHITECTURE?

Animal architectures do not build themselves. While the effect of selection might ultimately shape physical properties, selection itself must act on building behavior. A crucial link in understanding how animal architectures evolve is therefore understanding how they are built (Figure 2b). How does building behavior result in the architectures we observe and, crucially, how does variation in building behavior map onto variation in architecture?

Insects provide a useful model for addressing this question. In many insect species, building behavior is thought to be controlled by a process called “stigmergy” (Theraulaz & Bonabeau, 1999). Rather than individuals building by carrying out a stereotyped sequence of actions, in stigmergy the current state of the construction triggers the next appropriate behavior by the builders. This means that individuals can leave or join the building at any point and still exhibit the correct behavior for that stage of building, allowing work to be distributed across the colony. Stigmergy is thought to be controlled by behavioral rules: when the structure is in state X—perform action Y. These rules can therefore result in stages of construction, which continue until the state of the structure triggers a change in the rules which progresses construction onto the next stage. In mud wasps (*Paralastor* sp.), for example, nest tunnels are capped with funnel consisting of a tube‐like stem and a rounded bell made from mud pellets. The stem is constructed first and after the stem has reached a sufficient length, construction begins on the bell (Smith, 1978). By interrupting the wasps and changing the perceived state of the structure, it is possible to redirect construction, either keeping wasps within a particular stage of building, for example, burying the stem to decrease its perceived length, or even encouraging wasps to build a new funnel on top of an existing funnel by creating a hole in the top of the funnel when the wasps were close to finishing the structure. Although other insects, including paper wasps *Polistes fuscatus* (Downing & Jeanne, 1988, 1990) (Figure 1a), show more flexibility in the cues they use to control transitions between construction stages, stigmergy‐like behavioral rules provide a powerful mechanism for “unguided” building by insects, and by varying these rules it is possible to change the shape of the resulting structure. In the arid‐land subterranean termite, *Reticulitermes tibialis*, and the desert subterranean termites, *Heterotermes aureus*, species‐specific patterns of tunnel branching are the result of species differences in the proportion of individuals, either waiting to be at the front of the excavation queue or starting a new tunnel by excavating into the existing tunnel wall (Mizumoto et al., 2020). Species‐specific behaviors can also influence how a structure develops during construction. Both founder and worker termites of the long‐jawed desert termite, *Gnathamitermes perplexus*, and the subterranean desert termite exclusively remove debris during tunnel excavation by carrying particles in their mandibles. Founders and workers of the desert dampwood termite, *Paraneotermes simplicicornis*, on the other hand, can lift particles but usually remove debris by kicking it behind themselves. These differences in behavior influence the development of the tunnel: the kicking style of the desert dampwood termite initially results in a faster excavation than the carrying behavior of the long‐jawed or subterranean desert termites (Mizumoto et al., 2021). Over time, however, these patterns flip: with tunnel excavation by the dampwood desert termites slowing dramatically, while excavation rate by long‐jawed and subterranean desert termites remains constant or even increases.

Although vertebrate builders, such as nest‐ and tool‐making birds, are not thought to use stigmergy‐like rules (Walsh et al., 2013), documented variation in building behavior has still been linked to variation in the resulting structure. New Caledonian crows, *Corvus moneduloides*, for example, make and use tools to extract invertebrates from vegetation (Figure 1b). One particular type of tool, the “hooked stick tool,” is made by crows detaching a stick from a forked branch. Crows have been observed detaching the stick by either cutting it, pulling it, or both cutting and pulling (Sugasawa et al., 2017). These methods affect the shape of the tool as well as how well it functions. Cutting, for example, results in a deeper hook than pulling, and deeper hooks are quicker to extract bait (Sugasawa et al., 2017). Similarly, nests made by mature village weaverbirds, *Ploceus cucullatus*, are woven much more neatly compared to the first nests made by young birds, presumably reflecting the improvement of weaving skills through learning (Collias & Collias, 1964) (Figure 1c).

While these examples suggest a clear causal link between variation in behavior and variation in structure, simply inferring building behavior from structure is not straightforward. The desert dampwood termites, for example, produce a similar style of branching tunnels to the arid‐land subterranean termites, but achieve this using a completely different set of behavioral rules to those seen in either the arid‐land or the desert subterranean termites (Mizumoto et al., 2020). In these species, trying to infer behavioral similarity from physical similarity would be misleading: the two different structures are the result of slight tweaks to similar behaviors while the similar structures result from very different behaviors. If selection is acting on building behavior via the performance of the resulting structures, such apparent convergences of similar structures being produced from different behaviors might be expected. But if similar structures are produced by different behaviors, these differences could also act as a constraint on future evolution. Despite arriving at a similar structure, it is likely that variation in different building behaviors (e.g. debris‐kicking behavior and debris‐carrying behavior) would affect the physical properties of architectures differently. For example, an increase in one of these behaviors might make branching more likely than an increase in the other. It would be interesting to consider how behavior not only affects the current physical property (e.g. size, shape) of animal architectures, but also the “evolvability” of architectures when selection pressures change. Understanding these links between behavior, architecture, and evolution, however, requires a much more complete understanding of how behavior affects architecture than we currently have available.

## 3 HOW DO THE COSTS AND CONSTRAINTS ON BUILDERS AFFECT STRUCTURE?

Building does not happen in a vacuum. Just as structure is influenced by the behavior of the builders, the builders too are influenced by the environment and context in which they are working (Figure 2b). Behavioral ecology has a long history of examining behavioral evolution as the outcome of different costs, benefits, and constraints, and it is likely that this approach would be just as fruitful for examining the different pressures on building behavior. Building behavior can, for example, be constrained by the behavior of other individuals whose evolutionary interests might not line up with those of the focal animals, even individuals who are not involved with building at all. In stingless bees *Meliponinae*, for example, nests are composed of multiple brood cells, built by workers, into which the queen lays her egg (Figure 1d). The behavior of the queen differs between species, with the queens in species such as *Austroplebia symei* aggressively monopolizing newly built cells. This behavior is used by queens to control access to reproduction in species where there is a higher risk of workers laying their own eggs. As a result, builders in these species are only able to construct a single cell at a time, then waiting for the queen to provision and lay before moving on to build the next cell (Oldroyd & Pratt, 2015). This “one‐at‐a‐time” building style results in brood cells occurring in clusters. In contrast, in species such as *Plebeia saiqui*, where worker laying is rarer, there is weaker selection on queens to control access to the newly formed cells. This frees up workers to build multiple new cells simultaneously, resulting in regular combs or even spiral combs. Reproductive control in stingless bees therefore acts as a constraint on the evolution of nest architecture in these species, as selection on worker building behavior comes into conflict with selection on reproductive control by queens. Understanding how evolution has acted on architecture via behavior, therefore, requires more than just studying the morphology of structures, and even more than studying the behavior of builders. It also requires understanding the other selective pressures faced by builders and other individuals who have the potential to affect the process of construction.

Costs can also come from what builders could be doing instead of building. In New Caledonian crows, cutting sticks from branches results in deeper and better performing hooks than when crows pull sticks from branches (Sugasawa et al., 2017). Despite the apparent superiority of the cutting method, crows continue to use both cutting and pulling as alternative manufacture methods. The persistence of the pulling technique could be due to the costs and benefits associated with both the cutting and pulling methods. To detach a stick from the forked branch on the tool material, a crow must take two actions when starting the manufacture with cutting: either cut twice or cut and then pull. If the crow pulls the stick off of the branch, however, it can detach the stick in one action. In the latter case, the reduced efficacy of the tool might be offset by the time the crow saves for other behavior, such as foraging without a tool, being vigilant, or traveling. The case of the New Caledonian crows illustrates an important point: the best performing architecture might not always be the best option from the builder's perspective. In some cases, building a subpar architecture can be a better use of an animal's time than crafting something better. To gain the complete picture for how animal architecture contributes to fitness, it is therefore not sufficient to only look at how the final animal architecture performs, it is also necessary to look at building behavior and consider the potential balance of cost and benefit between alternative behavioral options. Doing so can provide opportunities to ask new questions about when and where different behavioral options are used.

## 4 HOW DOES LEARNING SHAPE BUILDING BEHAVIOR AND STRUCTURE?

Unlike morphology, behavior can change within an animal's lifetime, and be fine‐tuned to circumstances by learning (Figure 2b). The role of learning in avian nest building, for example, was suspected as early as the 19th century by the likes of Alfred Russell Wallace (Wallace, 1870). Since then, studies of nest building in birds have mostly assumed this behaviour to be innate (Guillette & Healy, 2015). In recent years, however, there is increasing evidence that birds learn various aspects of nest building, both in the lab experiments and in the wild (Breen et al., 2016). Several observations of nesting birds in the wild, for instance, concluded that birds that were unsuccessful at breeding were more likely to move to a new nesting site (Breen et al., 2016). Lab experiments, which enabled finer control over birds' experiences, have revealed that zebra finches *Taeniopygia guttata* can learn to adjust the way they hold nest material so that it fits through the entrance to the nest box (Muth & Healy, 2014), and will copy the choice of nest material from familiar conspecifics (Guillette et al., 2016) (Figure 1e). Similar effects of learning have been found in web building by the trashline orbweaver spider species *Cyclosa argenteoalba* (Nakata et al., 2003) and the missing‐sector orbweaver spider *Zygiella x‐notata* (Venner et al., 2000) (Figure 1f). These spiders relocate their webs, responding to several factors including prey availability, foraging frequency, and web damages induced by non‐prey items.

The role of learning in building behavior somewhat complicates the evolutionary story. While the feedback between building behavior, physical properties, and selection described above offers a framework for understanding how evolution can shape nest properties, it rests in part on the idea that building behavior is the product of selection. If nest properties are instead the product of animals modifying their behavior with experience, what exactly is being selected? Is selection only acting on “learning ability” or have animals also evolved learning biases or some other factor that results in nests adopting particular properties? In this sense, bird nests might be similar to bird songs. While species have a distinct song, there is still a well‐studied role for learning in this process (Hyland Bruno et al., 2021). Song learning can be biased by species‐specific templates for the learned song (Bolhuis & Moorman, 2015), by only paying attention to particular tutors (Soha & Marler, 2000), or the pre‐existing biases of receivers (Collins, 1999; Tencate & Rowe, 2007). Even in cases in which songs are entirely learned, song itself can still evolve via cultural changes, for example, if successful individuals are more likely to be copied than less successful individuals, resulting in a community converging on a particular song (Aplin, 2019; Whiten, 2019). Similar processes could guide the evolution of animal architecture, whether this is through individuals biasing learning based on inherited templates, or “species‐specific” designs being maintained through cultural rather than genetic evolution (Breen, 2021). Pulling apart the different contributions of learning and genetics is a complicated endeavor but could explain how the ability to fine‐tune behavior exists alongside selection for behavior imposed via physical properties.

## 5 DISCUSSION: ROUNDING OUT THE STUDY OF ANIMAL ARCHITECTURE

**FIGURE 3:**
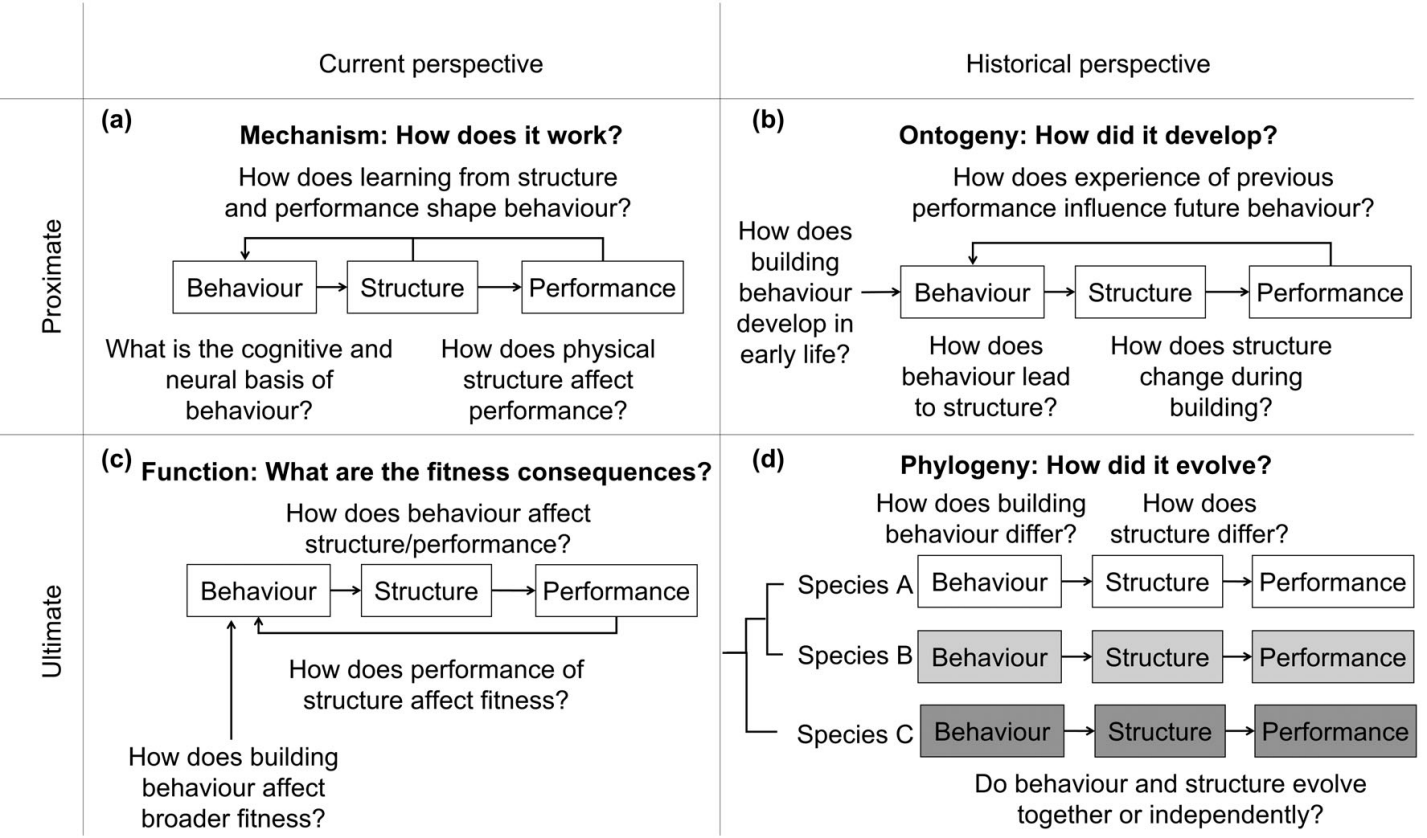
Applying Tinbergen's four questions to the study of animal architecture. Niko Tinbergen (1963) suggested four ways to answer the question of why an animal is performing a behavior: in terms of (a) the mechanism generating and controlling behavior, (b) the developmental history of the behavior, (c) the survival function of the behavior, and (d) the evolutionary history of the behavior. These questions provide a framework for studying both the proximate (mechanistic) and ultimate (evolutionary) causes of animal architecture across both short and long timescales, allowing questions of building behavior to be integrated alongside those addressing physical properties

Looking more at the role of building behavior in animal architecture does not mean ignoring architecture itself. The previous years of data show the value in looking at functional morphology of animal architectures (Hansell, 2005). Rather, as our examples above demonstrate, behavior has the potential to highlight patterns hidden in the apparently clear picture that studies of structure alone might provide. By looking at behavior alongside architecture, we can gain a more holistic view of how animal architecture is created and modified, both within the lifespan of an animal or structure, as well as across evolutionary time. To take inspiration from Niko Tinbergen's famous “four questions” framework for studying behavior (Tinbergen, 1963) (Figure 3), looking at behavior and structure together allows a more integrated understanding of how building behavior and animal structures work (mechanism), how building behavior and structures develop (ontogeny), the fitness benefits provided by building behavior and structure (function), and how building behavior and structures have evolved over time (phylogeny).

Consider, for example, the question of why birds use certain materials and not others to line their nests. Mechanistic questions about this could involve both questions about the decisions birds make when selecting material including the role for learning and memory (Bailey et al., 2016), as well as questions about how the architecture itself works, such as analyzing how the choice of material affects insulation properties (Dawson et al., 2011; Windsor et al., 2013) or reduces ectoparasite loads (Dawson et al., 2011; Gwinner et al., 2000). For both the behavior and the architecture, questions about function revolve around the effect of material on offspring recruitment (Järvinen & Brommer, 2020). But while functional questions about the architecture only consider how variation in nest material affects fitness, similar questions looking at behavior would not only look at the impact of behavior on the properties of the nest, but also consider the costs and benefits of carrying out that of behavior rather than an alternative.

Questions of function and mechanism address the “current” state of building behavior and the architecture, both in terms of how these are working and the benefits they offer. Questions of ontogeny and phylogeny, on the other hand, look into what happened in the past (Figure 3). In some cases, considering behavior alongside structure could clear up some current controversies. For example, several attempts to reconstruct phylogenetic trees based solely on avian nest characteristics have found varying degrees of mismatches with the birds' phylogeny (Winkler & Sheldon, 1993; Zyskowski & Prum, 1999). One explanation for this mismatch is that these studies are only looking at structure. As seen in the case of the termites mentioned above, the relationship between building behavior and the resulting structure might be less than straightforward. By explicitly looking at how variation in both structure *and* behavior maps onto phylogeny, we can gain a clearer picture for why species might produce different structures. Apparent homology in structure, for example, might disguise considerable variation in behavior, changing the perspective on how these structures actually came to resemble one another.

Finally, in order to examine animal architecture through the lens of Tinbergen's four questions, mechanisms, function, phylogeny, and ontogeny, it is critical to address Tinbergen's often‐forgotten fifth point: description (Tinbergen, 1963). In order to understand how animal construction works, how it develops, and how it evolves, it is first necessary to characterize what construction behavior actually looks like. Compared to descriptions of animal architectures, descriptions of building behavior are still relatively rare. This is almost certainly due to the difference in difficulty: while an animal‐built architecture is relatively simple to dissect and measure, behavior is transient, messy, and difficult to quantify. But advances in computational approaches are providing new opportunities for describing and quantifying behavior. Although in Tinbergen's time, the process of observations would have been heavily reliant on a notebook and a pencil, in recent years there has been an upsurge of free software that use deep learning to track target animals from footage (Günel et al., 2019; Mathis et al., 2018). They estimate the pose of the animal in each frame, enabling the quantification of building behaviors, such as “rubbing of moss” and “weaving of spider silk” that Tinbergen described (Tinbergen, 1953). For example, a recent study of orb spider building used open‐source deep‐learning tools to track the legs of cribellate orb weaver spider species *Uloborus diversus*. By looking at which kinds of movements occur together, they discovered that different stages of web‐building can be defined in terms of how likely spiders were to transition between a shared set of behaviors (Corver et al., 2021). New computational techniques are not just restricted to analyzing behavior, they can also assist in quantifying variation in architectures. Now that most smartphones have a good‐quality camera, for instance, photographs of structure images could be used to analyze material composition (Sugasawa et al., 2021) while specialized techniques like computerized tomography (CT) scan can provide cross‐sectional images of structures, revealing internal mechanical properties (Alba Tercedor et al., 2016). By better quantifying the nature and variation of animal architecture, we can provide a foundation for unraveling larger questions about how this fascinating interaction between animals and their environment is controlled, develops, and evolves.

## CONFLICT OF INTEREST

The authors declare that there is no conflict of interest.
